# Poly (safranine) modified carbon paste electrode as a sensor for the determination of fenitrothion pesticide

**DOI:** 10.1038/s41598-023-41498-3

**Published:** 2023-08-31

**Authors:** Ibrahim Shibl El-Hallag, Youssef Ibrahim Moharram, Sameh Mahmoud Selim

**Affiliations:** 1 Analytical and Electrochemistry Research Unit, Chemistry Department, Faculty of Science, Tanta University, Tanta, 31111 Egypt; 2 R&D and Microanalysis Laboratories, KZ for Pesticides and Chemicals Company, Nubaria City, Egypt

**Keywords:** Analytical chemistry, Electrochemistry

## Abstract

An effective electroanalytical method was developed for the quantitative evaluation of fenitrothion pesticide. The electrochemically modified carbon paste electrode CPE was constructed by applying a safranine layer on its surface. Safranine monomer is easily oxidized. So, a safranine layer was applied via electropolymerization using cyclic voltammetry in (2 × 10^–5^ M) safranine buffered solution with phosphate buffer BPS at pH.6, potential window (− 1.6: + 2 V), scan rate 100 mV/s. The morphology of the modified electrode was characterized using SEM images. The electropolymerization process was characterized by observing the gradual increases of the peak current with the subsequent scanning cycles. This modified CPE electrode showed an obvious sensitivity by cyclic voltammetry towards the cathodic peak of the fenitrothion nitro group at approximately − 0.73V with good sensitivity by enhancing it to be approximately 10 times more sensitive than on a bare carbon paste electrode CPE. The number of cycles was optimized for the electropolymerization process to be 12 cycles. Where, the peak current at − 0.73 V was gradually enhanced until 12 cycles when it is obviously decreased before slightly increasing again. The reproducibility of the modified electrode was ensured by repeating the sweep cycles using LSV for determining the fenitrothion at 5 µM where it was found that the peak current was unchanged for 10 sweeps before it starts to drop gradually. LSV voltammetry at previously optimized conditions of the potential window (− 0.4: − 1 V), sweep rate 100 mV/s, phosphate buffer at pH.6 was used for the quantitative studies. Where, the pHs of the determining medium were varied from pH 5.5 to pH 8 using phosphate buffer. It was observed that the most identified peak current was at pH.6 which is then decreased gradually until it completely disappeared at pH 8. The optimal accumulation time by adsorption of 140 s for the fenitrothion pesticide was confirmed in the range of (20 s–170 s). Where, the peak current was increased gradually with time up to 140 s then a plateau with a constant response was observed. The developed method showed an excellent linearity range of (1 μM:15 μM) with R^2^ parameter equal to 0.99906. LOD and LOQ were calculated to be 0.1 μM, and 0.34 μM respectively. Satisfactory levels have been reached for the calculated recovery, accuracy. Precision limits not exceed 1% for both repeatability and reproducibility measurements. F-value and t-value were measured for the suggested LSV method versus the standard HPLC method for the concentration of 8 μM fenitrothion and were found to be 1.482 and 0.123, respectively which didn’t exceed the tabulated values. The ruggedness of the suggested method was examined toward deliberate safranine concentration variations in the concentration range of (0.01 mM–0.03 mM). Insignificant differences for the mean recovery at (98.33–98.93%) and precision at (1.39–2.6%) were observed. Hence, the reliability and validity of the developed LSV method were achieved and it was considered as rigid method.

## Introduction

Fenitrothion is an organophosphate pesticide. It has been used as an insecticide and acaricide. Fenitrothion is used to eliminate and control agricultural pests. It is soluble in a wide range of organic solvents, and it has low solubility in the aqueous medium^1–3^. Fenitrothion is considered a short residual persistent pesticide in groundwater and soil. Therefore, it is classified as a moderately toxic pesticide for mammalians and highly toxic towards birds, honeybees, and aquatic living systems. The developed analytical methods used for quantification of fenitrothion were variable and different. The chromatographic technique was one of the most available and rapid techniques used in industrial and research work^4–6^. Usually, according to the standard methods of analysis, the fenitrothion pesticide is quantitatively determined by the LC technique. Alireza et al.^7^ used magnetic polymer as a sorbent for the extraction of fenitrothion. This polymer was fabricated using methacrylic acid as the monomer, ethylene glycol dimethacrylate as a cross-linker, and fenitrothion as the analyte. Fe_2_O_3_ nanoparticles were used as a magnetic support and then HPLC equipped with a UV detector was used for determining the fenitrothion. This last-mentioned adsorbent material showed an adsorbent maximum limit equal to 31.5 mg/g. The calibration curve was constructed for the developed method at a range of 0.3–50 μgL^−1^ with good linearity, and a LOD of 0.1 μgL^−1^. The precision measurements (RSD) at the 10 μgL^−1^ level, n = 5, for both repeatability and reproducibility were equal to 1.6%, and 3.1%, respectively. The use of conducting polymers has been and still is a major challenge in recent years to develop effective and promising analytical methods for detecting and quantifying of the organophosphate pesticides. Melis kesik et al.^8^ fabricate a promising biosensor for detecting of organophosphate pesticides based on a conducting polymer on the surface of the modified graghite electrode. Where the conducting polymer (poly (SNS-NH2)) was applied on the surface of the working electrode via the electropolymerization process then Acetylcholinesterase (AChE) was successfully linkage by covalent bond on its surface. The inhibition effect of parathion, paraxone, and chlorfenvinphos pesticides toward the (AChE) enzyme on the surface of the modified electrode was investigated. Good results with a wide linearity range of (0.05 mM–8 mM), LOD of 0.09 mM, and high sensitivity according to the extremely high surface area of the modified electrode with the conducting polymer. Imran Khan et al.^9^ used the electropolymerization technique for the application of polyaniline layer as a conducting polymer on the surface of GCE and then the silver nanoparticles and multiwalled carbon nanotubes were electrochemically applied to this previously pretreated surface of GCE with polyaniline. This sensor was fabricated to determine the fenitrothion in real samples with good recoveries in between 95.2% and 100.04%. The quantification studies were performed using nonelectrolytic (adsorptive) deferential pulse stripping voltammetry (AdDPSV), and cyclic voltammetry (CV). The developed method using the previously modified GCE electrode showed very good linearity with an R^2^ statistic parameter equal to 0.998 in the range from 0.1 to 1.5 μg mL^−1^. Since the discovery of polyacetylene in 2000 by Shirakawa et al.^10^ and receiving the Nobel Prize in chemistry this year. Discovering, and improving the properties of various applications of conducting polymers has become a promising challenge for scientists. Awuzie, C^11^, ensures that the electrical transport properties of the conducting polymers can be controlled and improved by adjusting the parameters of electropolymerization. Thus, in the current work, the optimization methodology was followed to obtain an electrocatalytically active polysafranine layer on the surface of a carbon paste electrode (CPE) for fenitrothion pesticide sensing. Electropolymerization is considered as one of the most effective, easy, fast, and time-saving methodologies for applying the conducting polymers on the surface of the working electrodes. This should be performed using easily oxidized aromatic monomers such as aniline, pyrrole, and safranine. The electropolymerization process may be performed using potentiostatic techniques such as chronoamperometry or potentiodynamic techniques such as cyclic voltammetry. In this work cyclic voltammetry CV was adopted to apply the poly safranine layer on the surface of CPE. This layer will improve the catalytic conducting ability of the electrode towards fenitrothion sensing and increase the surface area which enhances the adsorption ability of the modified electrode later^12^. Salsabeel Al-Sodies et al.^13^ made a good review about the sensing applications of poly safranine and poly luminol in the chemical and biochemical field. Where, they reviewed the study of xy liu et al.^14^ about constructing a sensor for detecting para nitro phenol (4NP) in water samples. The poly safranine was applied on the surface of GCE then the cyclic voltammograms in between (− 1.6 to + 2 V) were recorded which indicate reduction peak of (4NP) at − 1.05 V. depending on this 4NP reduction peak the linearity was confirmed in the range confined in (0.08 µM- 40 µM) and limit of detection equal to 0.03 µM of (4NP). Caffeine was one of the substances that has been determined using electroanalytical methods. Where guo et al.^15^ applied safranine layer on GCE via electropolymerization in phosphate buffer at pH 5.8 using cyclic voltammetry for 20 cycles in between (− 0.8 V to + 1.2 V) then this polymer layer was coated with Nafion solution. The activity of this conducting polymer toward a known concentration of caffeine was examined at 10µM which detected by the anodic peak at 1.38 V. This anodic peak was raised gradually by increasing the caffeine solution concentrations ensuring the good fitted linear relationship with limit of detection equal to 0.1 µM. D. Saritha et al.^16^ constructed modified CPE electrode by drop casting NiO nano material on the surface of the CPE, then they applied the layer of the polysafranine conducting polymer via the electropolymerization process. This composite was used successfully to detect rutin flavonol glocoside compound. Authors used cyclic voltammetry and differential pulse voltammetry to validate a promising test method using this last-mentioned novel sensor of CPE modified with (NiO nano particles / polysafranine conducting polymer). The sensor showed good sensitivity, detection, and quantification ability towards the target analyte in the concentration range of (0.016 µM–0.23 µM). LOD at 0.0054 µM and LOQ at 0.016 µM were calculated successfully for the developed method. The rutin compound was determined in the green tea with good recoveries confined in the range of (96.5–102.8%). One of the most important application expected for the current study is the clinical application. This study may be considered as the paradigm for using safranine-O biological stain which is already used in the field of histology and cytology, in the form of conducting polymer to accumulate the fenitrothion pesticide molecules from the human cell using a promising therapy method. Depending on the high surface area character of the conducting polymer, the poly safranine may act as a precursor competitor for the cholinesterase enzyme. It binds with the fenitrothion molecule and inhibits the cholinesterase enzyme work in the human body. This will require more efforts and partnership between the basic science researchers and clinicians as it was done in many previous translation medicine studies^17,18^.

## Materials and methods

### Reagent and solutions

Analytical grade of fenitrothion 98% purchased from Sigma Aldrich- Germany used without further purification. Aceton HPLC grade, Safranine O stain for microscopic uses from Loba Chemia–India, Phosphoric acid is 85% from Oxford–India, potassium phosphate monobasic is 99.5%, potassium phosphate dibasic is 99% from Alpha Chemika–India, graphite powder ˂ 20μm extra pure is from Merck–Germany, paraffin oil for IR spectroscopy is from Loba–India, and deionized water has been obtained from double glass distilled water apparatus (Stuart-England). 1M stock solution of potassium phosphate monobasic as solution 1 was prepared, then 1M stock solution of potassium phosphate dibasic was used as a solution 2 was prepared. Therefore, 0.1M phosphate buffers at various pHs were prepared by adding calculated quantities from solution (1) to solution (2). 0.02 mM safranine solution was prepared by weighing 7.01 mg of safranine green technical powder and diluted with phosphate buffer solution (BPS) pH.6 in a 1L volumetric flask. Then this solution was used to construct the conducting polymer layer on the surface of CPE using cyclic voltammetry (CV) (sequential cycles) to obtain an electropolymerized safranine conducting layer. For the pretreatment of CPE before electropolymerization 5 cycles of cyclic voltammetry were done using 0.1M phosphoric acid solutions. A stock standard solution of fenitrothion was prepared in acetone and stored in the refrigerator, and then an aqueous solution was prepared by dilution of this stock solution with 0.1M phosphate buffer solution pH.6 to obtain the desired fenitrothion concentrations.

### Instrumentation

Potentiostat model 273A-PAR (Princeton applied research, Oak Ridge, TN, USA) equipped with a C-2 stand and magnetic heating stirrer. Operation software 270/250-PAR was used. HPLC system, series 200, (Perkin-Elmer, USA) equipped with an LC pump with maximum pressure of 6100 psi, Anion-exchange 10 SAX column, and a UV–VIS detector. pH meter (Ohaus-AB23PH-F, USA). Silver/ silver chloride (Ag/AgCl) 3M KCl reference electrode, platinum wire counter electrode, and micropipette for preparing different concentrations of fenitrothion.

### Construction of the modified CPE using safranine by electropolymerization

**Figure 1:**
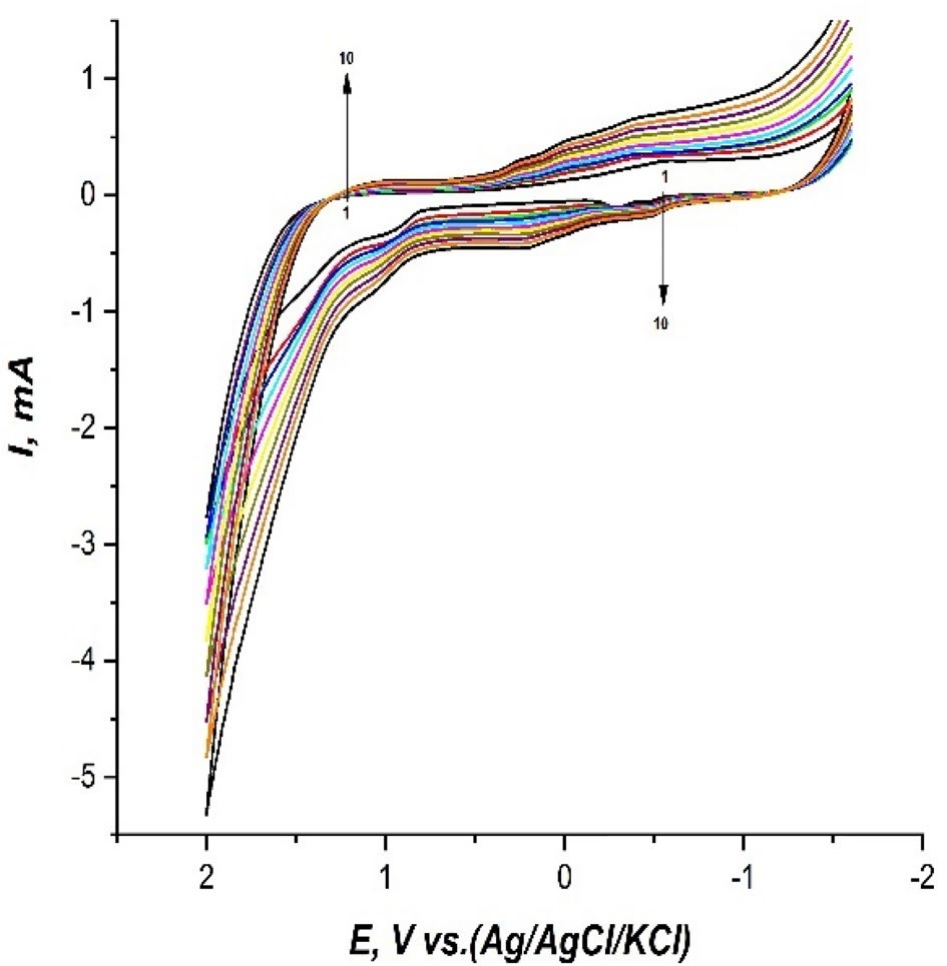
Cyclic voltammograms without stirring in a 10 ml electrolysis cell containing 0.02 mM safranine in BPS pH.6 solution, scan rate 100 mV/s, and potential window (− 1.6:+ 2 V), of subsequent 12 previously optimized cycles.

The carbon paste was prepared by homogenizing 5 g graphite fine powder with 1.9 ml paraffin oil in the mortar. Then CPE was firmly packed with this carbon paste at the end cavity then it was polished to a mirror finish with polishing paper (butter paper). A pretreatment with a 0.1M phosphoric acid solution was performed using cyclic voltammetry (5 cycles) in order to clean the surface from any possible contaminants and for smoothing the electrode surface. The CPE was immersed in a 10 ml electrolysis cell containing 0.02 mM safranine in BPS pH.6 solution, and then the electropolymerization was performed by recording 12 cyclic voltammograms without stirring of previously optimized at a scan rate 100 mV/s, with in a potential window of (− 1.6:+ 2 V). This modified CPE with an electrodeposited polysafranine layer was used directly in fenitrothion quantitative studies and showed reliable reproducibility. It was observed that intensities of the peak currents increase with increasing the subsequent scanning cycles and this may be attributed to the deposition of safranine aromatic monomer on the surface of CPE to form the polysafranine as conducting polymer. This gradually increasing peak current phenomenon proved the electropolymerization process as shown in the Fig. 1.

**Figure 2:**
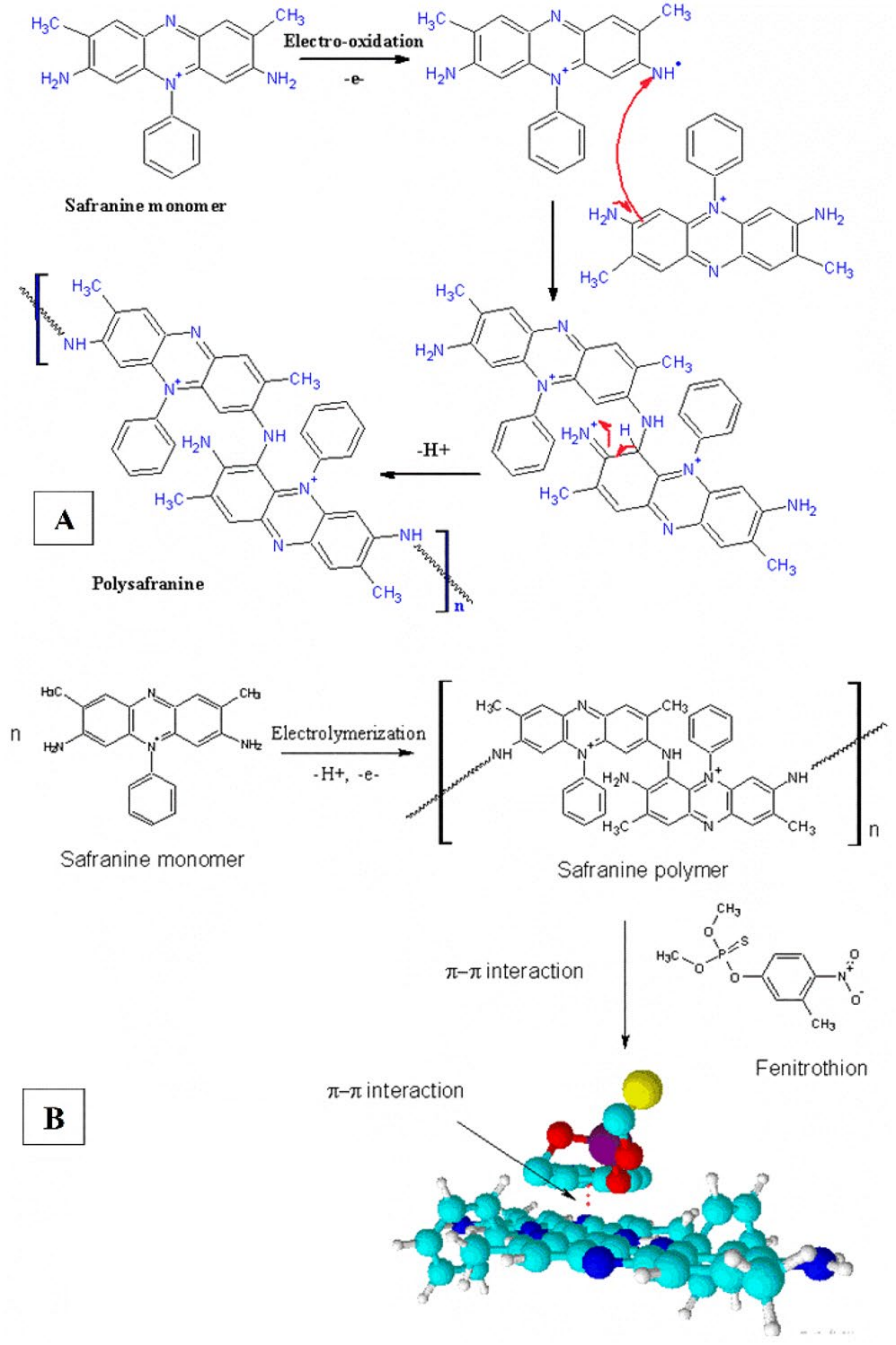
(**A**) The mechanism of the electropolymerization of safranine aromatic monomer on the surface of CPE, (**B**) the suggested mechanism of the interference of the polysafranine with the fenitrothion analyte on the modified CPE interface.

The mechanism of the electropolymerization process was elaborated as shown in Fig. 2A.

Where the Safranine monomer undergoes electrooxidation by losing one electron from one of the two amino groups leading to the formation of radical cation. After that another molecule of safranine work as nucleophile by attacking the electron deficient amino group in the previously oxidized monomer. After that the first dimer formed by proton elimination accompanied rearrangement to attain the ring aromaticity. The formation of polysafranine occurs through repeating this step. The suggested mechanism for the interaction or the crosslinking between the poly safranine and the fenitrothion analyte was stated as shown in Fig. 2B.

The polysafranine has two active centers which are available for interaction (free amino group through hydrogen bonding and aromatic structure through π–π bonding). On the other hand, fenitrothion has (sulfur group which is available for hydrogen bonding and a benzene ring which can form π–π bond). However, the amino group which is well known as active center for hydrogen bonding is not exposed for the interaction due to the crowded structure. Therefore, we suggest that the polysafranine interacts with fenitrothion molecule through π–π interaction^19^. This in addition to the high porosity of the conducting polymer layer which increases the surface area hence increasing the adsorption of the analyte on the electrode interface thus increasing the sensitivity of the modified CPE.

**Figure 3:**
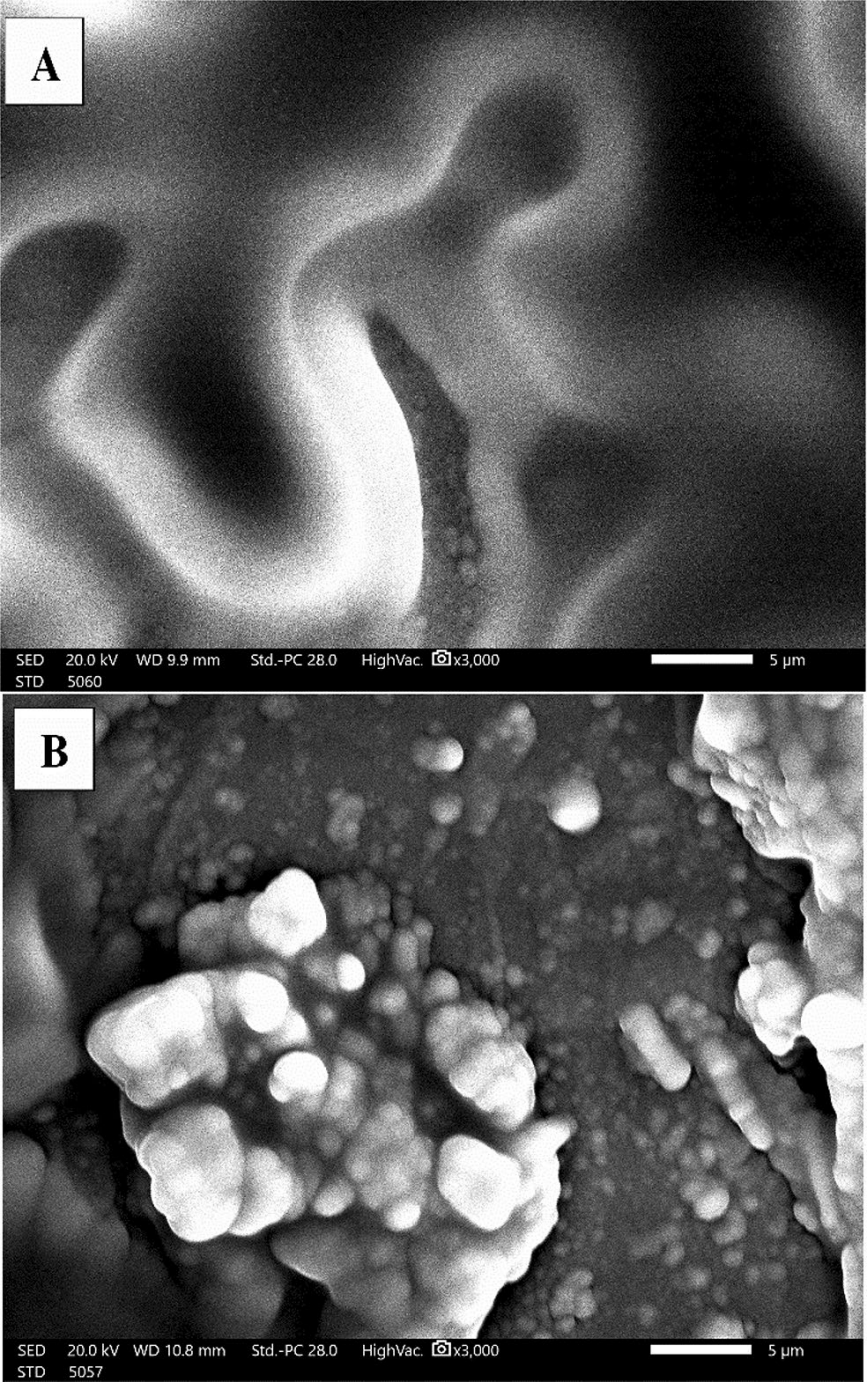
(**A**) SEM image of the surface of the bare CPE before the electropolymerization of safranine monomer. (**B**) SEM image of the surface of the modified CPE after the electropolymerization in 0.02 mM safranine solution in pH.6.

The morphology of the modified electrode was investigated using scanning electron microscope. By matching (SEM) images Fig. 3A,B. The polymer growth was proven on the surface of the CPE as shown in Fig. 3B.Which in turn confirms the success of the electropolymerization process on the surface of the modified electrode. This in addition to the gradual increasing phenomenon of the peak current as shown in Fig. 1, which proves and characterizes the electropolymerization process.

### Electrochemical procedures

The CPE was carefully packed with the carbon paste then it was polished, treated, and modified using the electropolymerization technique by safranine aromatic monomer, as last mentioned in the previous section. The modified CPE was immersed in different concentrations of fenitrothion in PBS pH.6, and the cyclic voltammograms were recorded with and without fenitrothion, scan rate 100 mV/s with a potential window of (+ 0.6: − 1.3 V). Cyclic voltammetry (CV) as a characterization technique showed a reliable cathodic peak for the fenitrothion at − 0.73 V which showed linear behavior with the fenitrothion concentrations, so this peak was used later for quantitative studies by linear sweep voltammetry (LSV) as a potentiodynamic technique. For quantitative measurements, LSV was used at a sweep rate of 100 mV/s, pH.6, a potential window (− 0.4: − 1 V), and optimized accumulation time of 140 s. Then the linear sweep voltammograms were recorded without and with various concentrations of fenitrothion with reliable reproducibility of the modified electrode.

## Results and discussion

**Figure 4:**
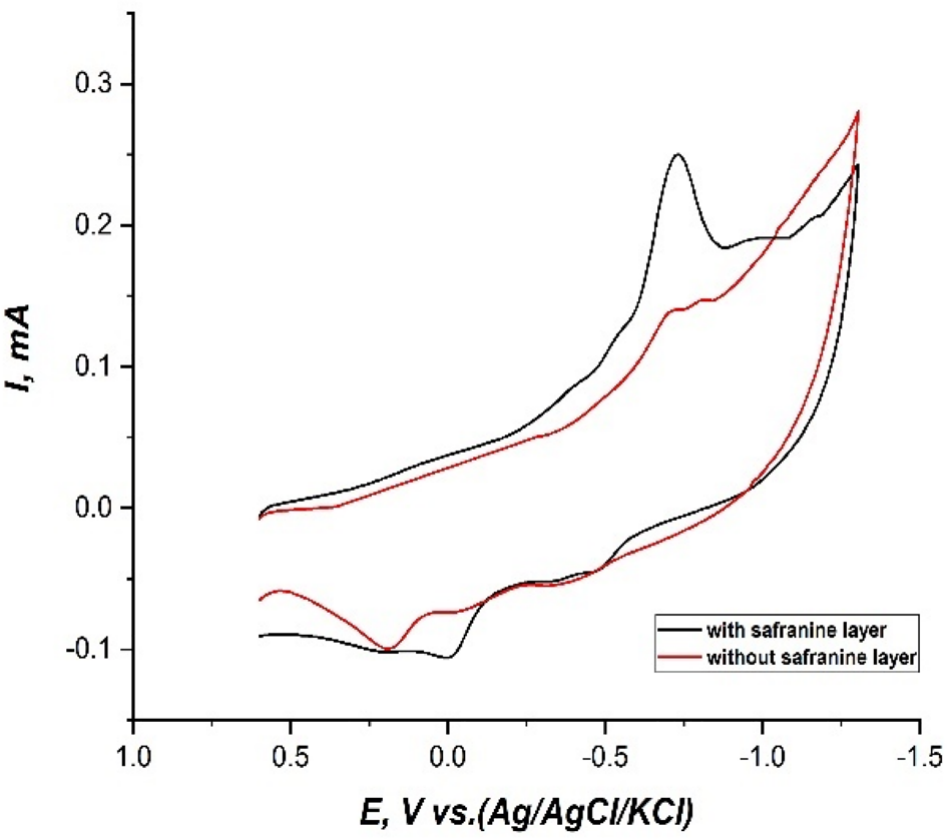
Cyclic voltammograms of 3 × 10^–5^ M fenitrothion in 0.1M phosphate buffered solution at pH.6, scan rate 100 mV/s, (+ 0.6: − 1.3V), equilibrating time: 25 s, on bare CPE and modified CPE with safranine by electropolymerization.

In this work, cyclic voltammetry (CV) as a characteristic tool was used to study the electrochemical behavior of fenitrothion on the modified CPE and the bare CPE. Cyclic voltammograms of 3 × 10^–5^ M fenitrothion in a 0.1M phosphate buffered solution at pH.6 on bare CPE and modified CPE with (safranine by electropolymerization) were recorded. The abroad and small reduction peak (Pc1) at the cathodic direction appeared approximately at − 0.73V on the bare CPE. This peak may be attributed to the reduction of the amino group of fenitrothion pesticide to hydroxyl amine. Then this hydroxyl amine group is reoxidized into the nitroso group with an identified peak (Pa1) at + 0.18V. This cathodic peak (Pc1) at − 0.73 V was obviously enhanced on the modified CPE with safranine monomer by electropolymerization as shown in Fig. 4.

**Figure 5:**
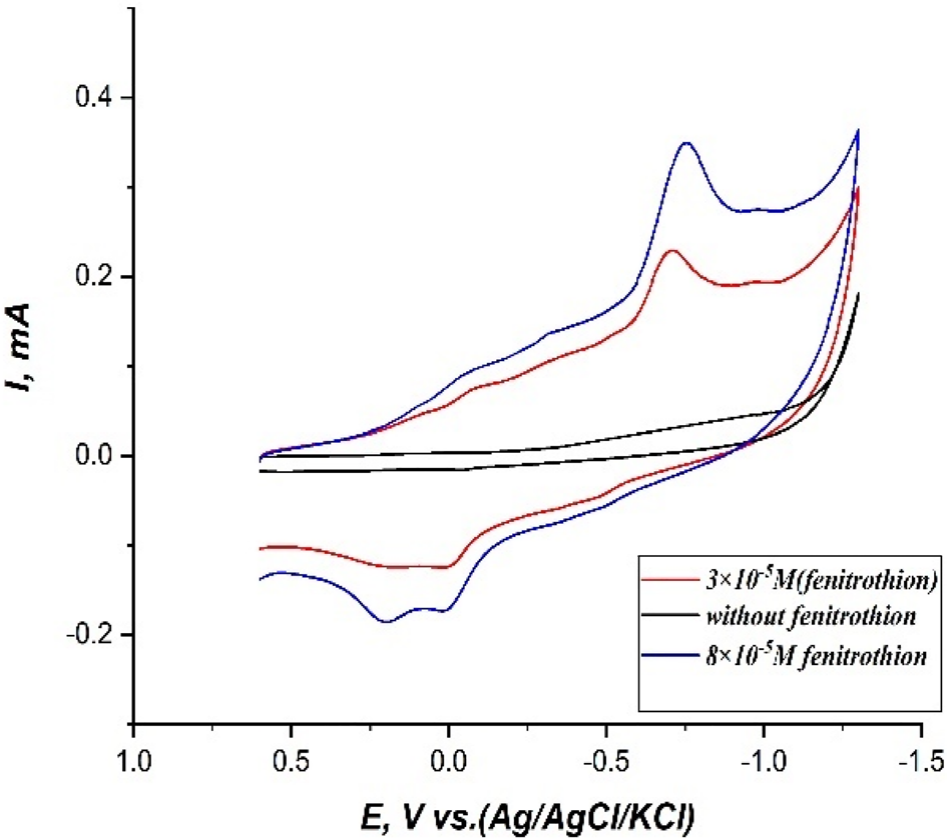
Cyclic voltammograms of the modified CPE with safranine in the presence and absence of different concentrations of fenitrothion in a 0.1M phosphate buffered solution at pH.6, scan rate 100 mV/s, (+ 0.6:− 1.3V), equilibrating time: 25 s.

This may be attributed to the catalytic impact of the modified CPE where the conducting polymer serves the electron transportation at the working electrode interface. Furthermore, the high surface area of the modified CPE enhances the adsorption of the fenitrothion molecules on the working electrode interface. So accordingly, the peak current has been enhanced by adding the fenitrothion analyte gradually as shown in Fig. 5.

### Optimization using cyclic voltammetry

#### Effect of the cycle’s number on the nature of the safranine- construct layer

**Figure 6:**
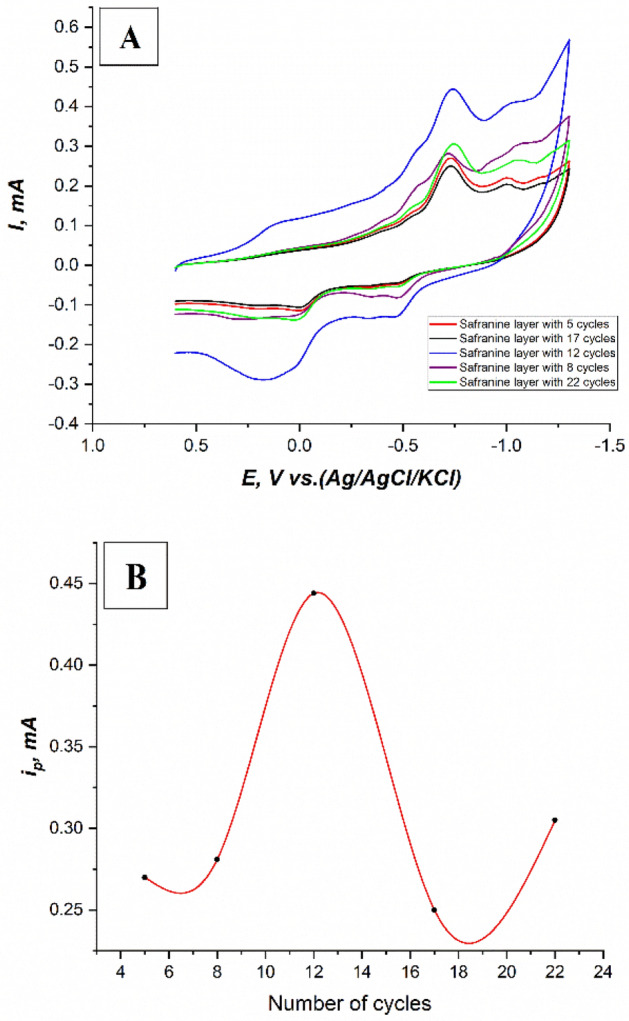
(**A**) Cyclic voltammograms of 3 × 10^–5^ M fenitrothion on (the modified CPE with safranine 5, 8, 12, 17, and 22 cycles in the construction step), in 0.1M PBS at pH.6, scan rate 100 mV/s, and potential window (+ 0.6: − 1.3 V), (**B**) A plot of (number of cycles-i_p_) for the corresponding cyclic voltammograms of 3 × 10^–5^ M fenitrothion on (the modified CPE with safranine (5, 8, 12, 17, and 22 cycles in the construction step), in 0.1M PBS at pH.6, scan rate 100 mV/s, and potential window (+ 0.6: − 1.3 V).

The effect of changing the number of cycles on the nature of the electropolymerized safranine layer have been studied using cyclic voltammetry. Cyclic voltammograms of 3 × 10^–5^ M fenitrothion on the modified CPE with safranine aromatic monomer (5, 8, 12, 17, and 22 cycles) in 0.1M PBS at pH.6, scan rate 100mV/s, and potential window (+ 0.6: − 1.3 V) have been recorded. It was noticed that the peak current at − 0.73 V is gradually enhanced until 12 cycles when it is obviously decreased before slightly increasing again as shown in Fig. 6A and the corresponding Fig. 6B. The most identified peak current was recorded at − 0.73 V, and by 12 cycles. Therefore, the constructed safranine layer was adopted by 12 cycles for constructing the modified CPE.

#### Effect of pHs. Value of phosphate buffer solution (PBS)

**Figure 7:**
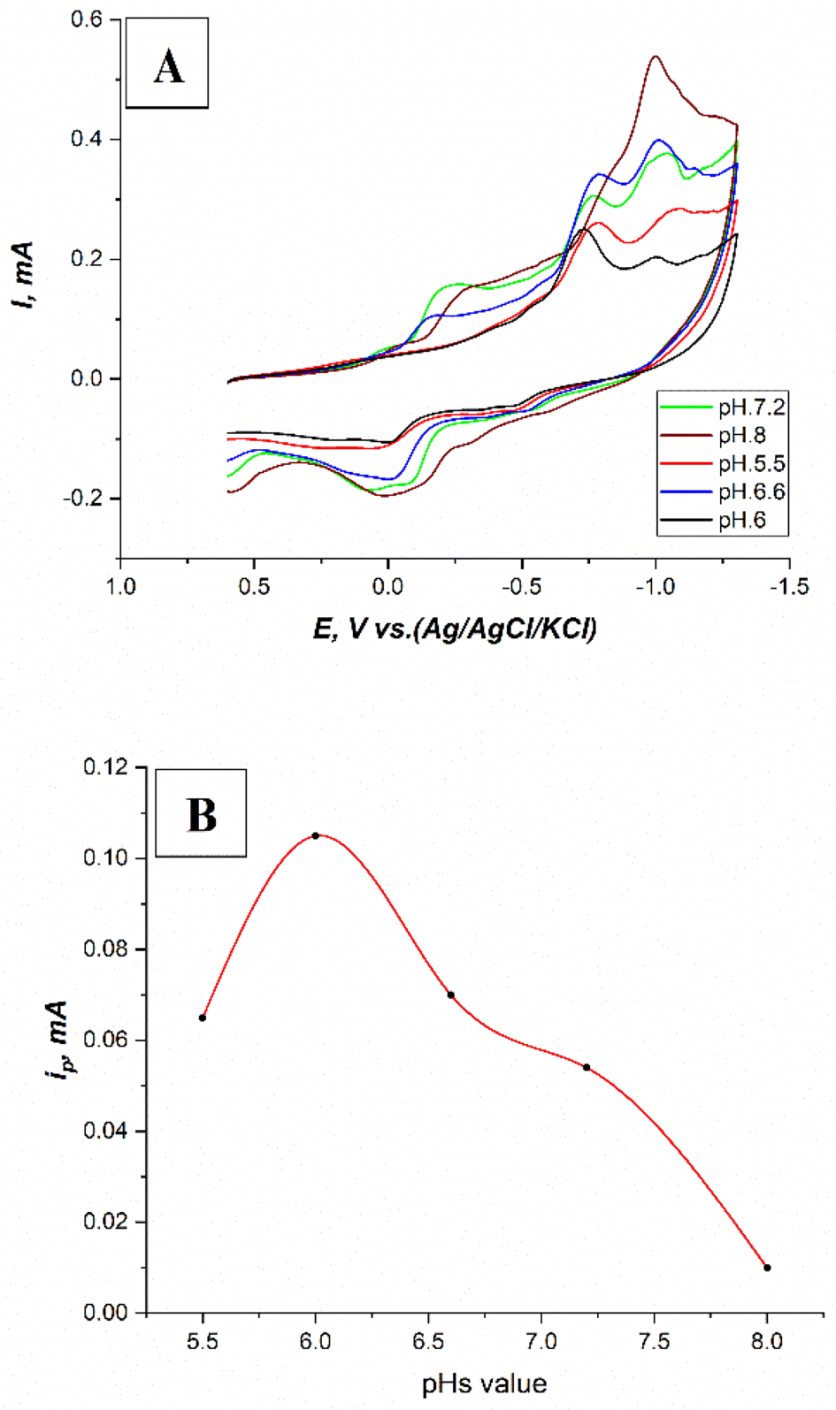
(**A**) Cyclic voltammograms of 3 × 10^–5^ M fenitrothion on (the modified CPE with safranine layer, 12 cycles), in 0.1M PBS at different pH values equal to (5.5, 6, 6.6, 7.2, and 8), scan rate 100mV/s, and potential window (+ 0.6: − 1.3 V), (**B**) A plot of (i_p_–pH_s_) for the corresponding cyclic voltammograms of 3 × 10^–5^ M fenitrothion on (the modified CPE with safranine layer, 12 cycles), in 0.1M PBS at different pH values, scan rate 100 mV/s, and potential window (+ 0.6: − 1.3 V).

A phosphate buffer solution was adopted for use in this current study^20,21^. Cyclic voltammograms of 3 × 10^–5^ M fenitrothion on (the modified CPE with safranine layer, 12 cycles), in 0.1M PBS at different pH values equal to (5.5, 6, 6.6, 7.2, and 8), scan rate 100mV/s, and potential window (+ 0.6: − 1.3 V) have been recorded. It was observed that the most identified peak current was at pH.6 as shown in Fig. 7B, and then it decreased gradually until it completely disappeared in an alkaline medium. This may be attributed to the deprotonation phenomenon in the alkaline medium for the hydroxyl amine group thus enhancing the formation of the azoxy group combined with the gradually disappearance of the reduction peak of the nitro group at − 0.73 V, and the appearance of another peak at − 1 V as shown in Fig. 7A. Therefore, the phosphate buffer solution at pH.6 was chosen as the optimal pH value for this work.

Optimization of scan rate was performed in the determining step section using linear sweep voltammetry (LSV). It was found that the most consistently identified peak current was 100 mV/s as shown in the section of quantitative studies using (LSV).

#### Effect of different concentrations of fenitrothion pesticide using cyclic voltammetry

**Figure 8:**
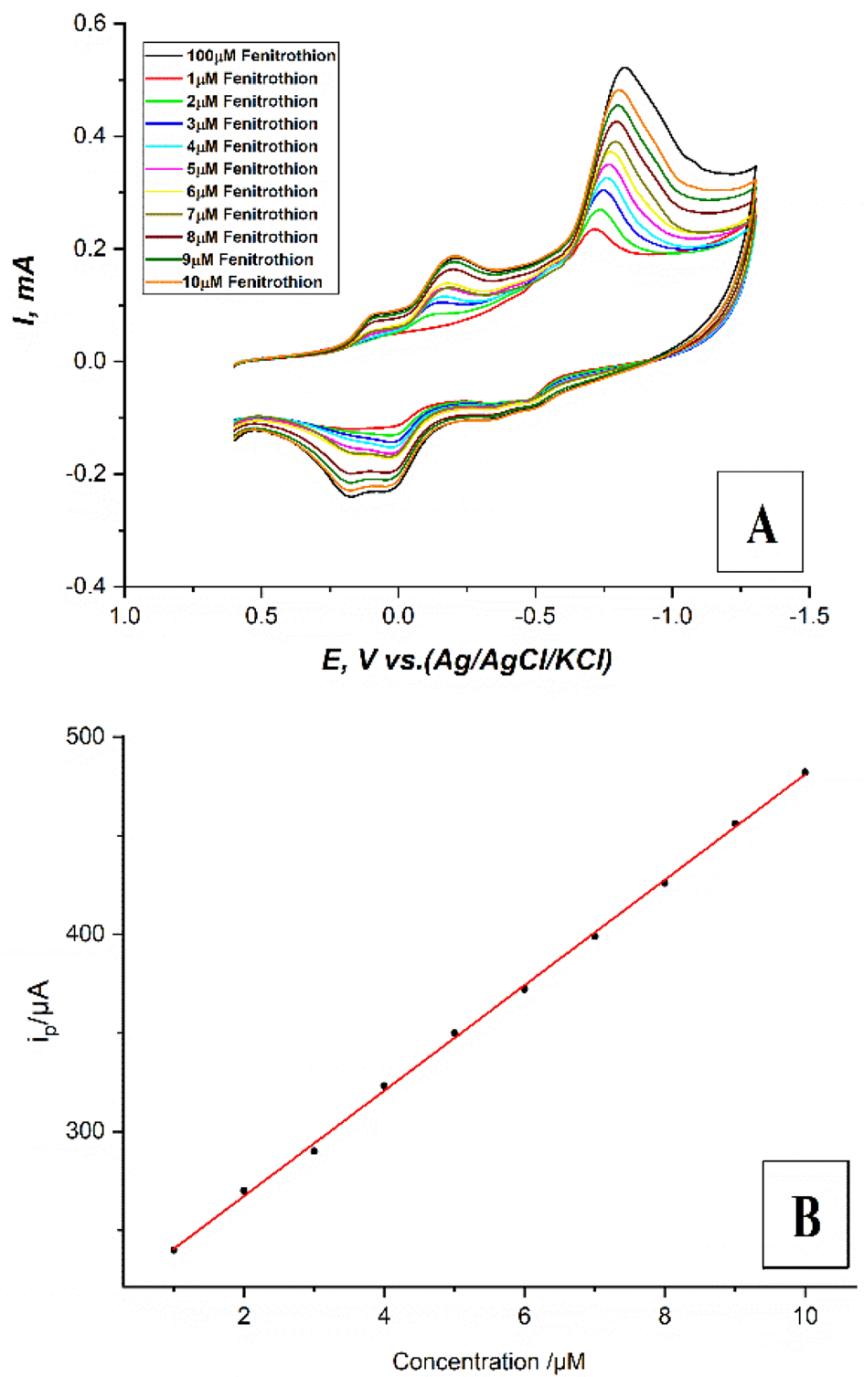
(**A**) Cyclic voltammograms for different concentrations of fenitrothion pesticide in the range (1–10 μM), on (the modified CPE with safranine layer, 12 cycles), in 0.1M PBS at pH.6, scan rate 100mV/s, and potential window (+ 0.6: − 1.3 V), (**B**) A plot of (i_p_—fenitrothion concentrations) for different concentrations of fenitrothion pesticide in the range (1–10 μM), on (the modified CPE with safranine layer, 12 cycles), in 0.1M PBS at pH.6, scan rate 100 mV/s, and potential window (+ 0.6: − 1.3 V).

The electrocatalytic ability of the modified carbon paste electrode CPE with safranine aromatic monomer via the electropolymerization process was demonstrated by the reduction of different concentrations of fenitrothion pesticide in the range (1 μM–10 μM). Cyclic voltammograms for different concentrations of fenitrothion pesticide in the range (1 μM–10 μM), on (the modified CPE with safranine layer, 12 cycles), in 0.1M PBS at pH.6, at a scan rate 100 mV/s, and in the potential window (+ 0.6: − 1.3 V) have been recorded. Regular increases in the reduction (cathodic) peak current of the nitro group at − 0.73 V were noticed as shown in Fig. 8A. This may be attributed to the conducting polysafranine layer which enhances electrons transportation at the working electrode interface. Also, the high porosity of this conducting polymer layer makes the surface area of the working electrode is too large. This high surface area enhances the adsorption of fenitrothion molecules on the surface of the modified CPE resulting in an increase in the peak current.

The corresponding Fig. 8B shows excellent linearity with an R^2^ parameter equal to 0.99915 and this was the paradigm to use linear sweep voltammetry (LSV) as rapid and more selective potentiodynamic technique to perform the validation for the suggested developed method.

### Quantitative studies using the LSV potentiodynamic technique

#### Optimization of accumulation time

**Figure 9:**
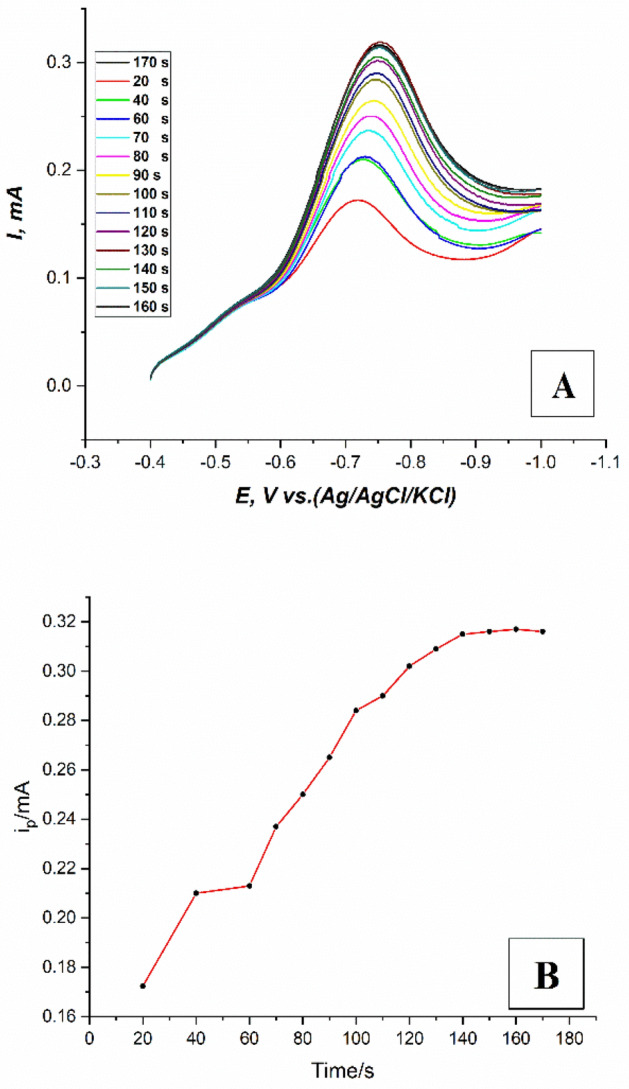
(**A**) LS voltammograms of 3 × 10^–6^ M fenitrothion on (the modified CPE with safranine layer, 12 cycles), in 0.1M PBS at pH.6, sweep rate 100mV/s, and potential window (− 0.4: − 1 V) at different accumulation time, (**B**) A plot of (i_p_−T) for LS voltammograms of 3 × 10^–6^ M fenitrothion on (the modified CPE with safranine layer, 12 cycles), in 0.1M PBS at pH.6, sweep rate 100mV/s, and potential window (− 0.4: − 1 V).

The effect of accumulation time by adsorption was determined by recording the LS voltammograms of 3 × 10^–6^ M fenitrothion on (the modified CPE with safranine layer, 12 cycles), in 0.1M PBS at pH.6, sweep rate 100 mV/s, and potential window (− 0.4: − 1 V) as shown in Fig. 9A. The peak current was increased gradually with time up to 140 s then a plateau with a constant response was observed as shown in Fig. 9B. This may be attributed to the maximum coverage of the surface of the working electrode by adsorption with fenitrothion molecules. Therefore, the accumulation time by adsorption of 140 s was adopted as the optimal time in the LSV developed method.

#### Effect of scan rate

**Figure 10:**
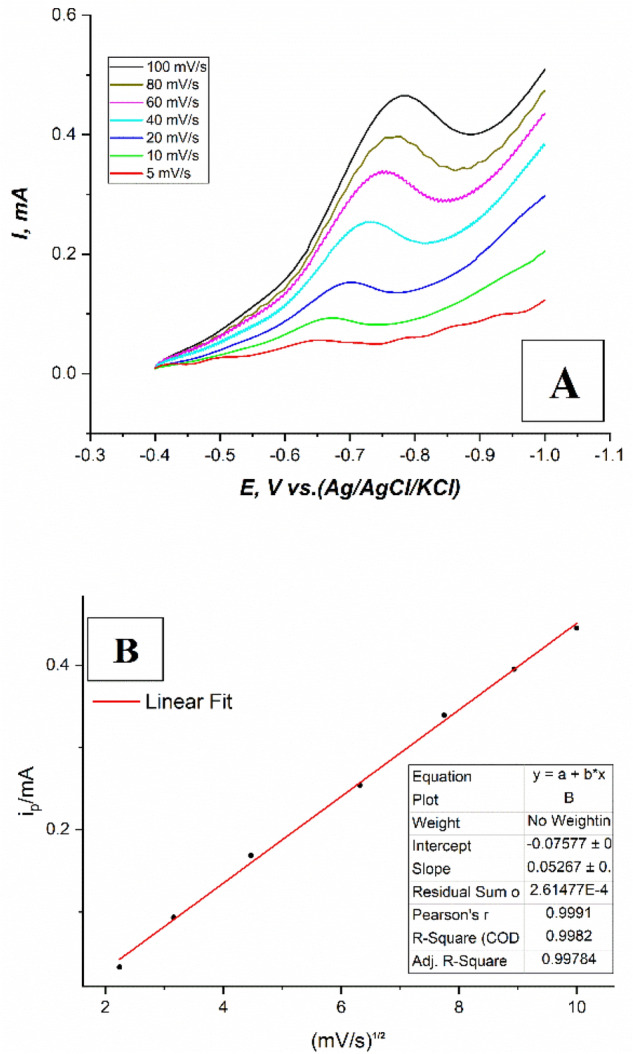
(**A**) LS voltammograms of 8μM fenitrothion solution on the modified CPE, in 0.1M PBS at pH.6, at different sweep rates (5, 10, 20, 40, 60, 80, 100 mV/s), and potential window (− 0.4: − 1 V), (**B**) A plot of (i_p_ vs. square root of scan rate) for linear sweep voltammograms LSV of 8 μM fenitrothion solution on the modified CPE, in 0.1M PBS at pH.6, at different sweep rates (5, 10, 20, 40, 60, 80, 100 mV/s), and the potential window (− 0.4: − 1 V).

The effect of scan rate on the reduction peak current of the nitro group of fenitrothion was investigated in 8 μM fenitrothion solution on the modified CPE, in 0.1M PBS at pH.6, at different sweep rates (5, 10, 20, 40, 60, 80, 100 mV/s), and in a different potential window (− 0.4: − 1 V) using the LSV suggested method. It was observed that the peak current was enhanced gradually by increasing the scan rate as shown in Fig. 10A.

The relation between the peak current and the square root of the scan rate was constructed and a well fitted linear relationship with the R^2^ parameter equal to 0.9982 was reached which proves that the process is under diffusion control as shown in the Fig. 10B^22–24^.

**Figure 11:**
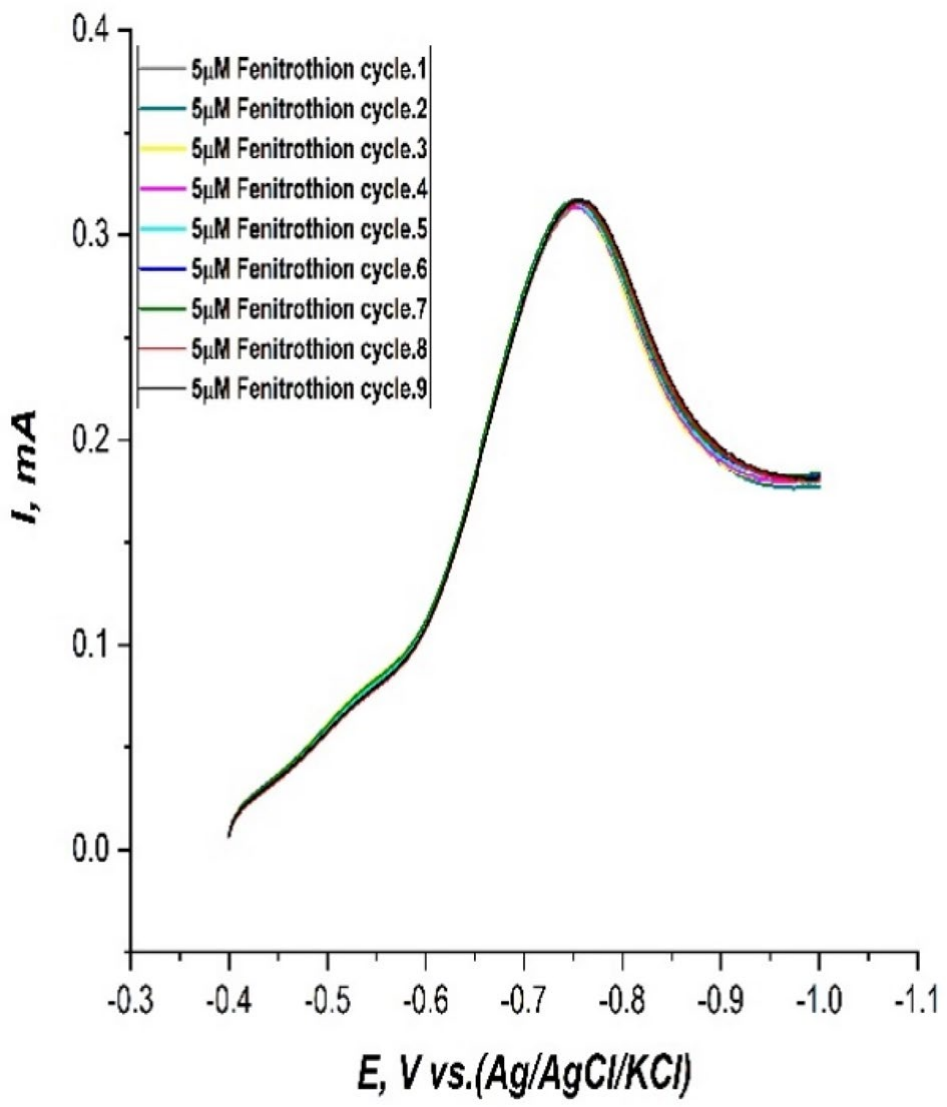
LS voltammograms of 5µM fenitrothion solution 10 time repeated on the modified CPE, in 0.1M PBS at pH.6, sweep rate of 100 mV/s, and potential window (− 0.4: − 1).

At all of these previously optimized method parameters the stability and reproducibility of the fabricated sensor was experimentally confirmed. 10 time repeatedly LS voltammograms of 5 µM fenitrothion solution on the modified CPE, in 0.1M PBS at pH.6, sweep rate of 100 mV/s, and potential window (− 0.4: − 1) were recorded. It was found that the peak current was unchanged for 10 time as shown in Fig. 11 before it starts to drop gradually. This was evidence for the reproducibility and stability of the fabricated CPE.

#### Linearity range, LOD, and LOQ calculations

**Figure 12:**
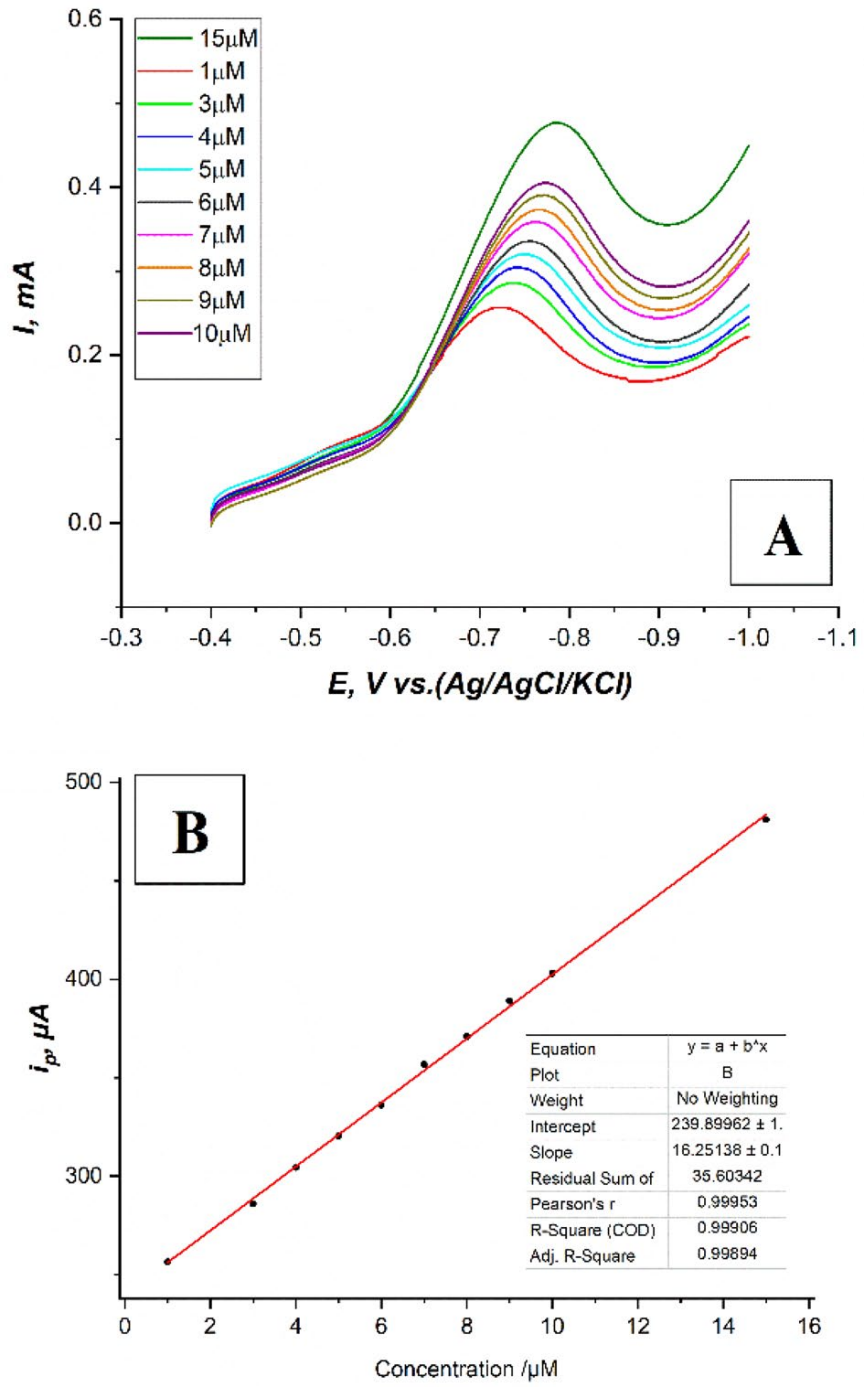
(**A**) Linear sweep voltammograms LSV of different concentrations of fenitrothion pesticide in the range of (1–15 μM), on (the modified CPE with a safranine layer, 12 cycles), in 0.1M PBS at pH.6, sweep rate 100mV/s, accumulation time equal to 140 s, and potential window (− 0.4: − 1 V), (**B**) A plot of (i_p_ vs. fenitrothion concentrations) for Linear sweep voltammograms LSV of different concentrations of fenitrothion pesticide in the range of (1–15 μM), on (the modified CPE with safranine layer, 12 cycles), in 0.1M PBS at pH.6, sweep rate 100 mV/s, accumulation time equal to 140 s, and potential window (− 0.4: − 1 V).

Using previously optimized conditions linear sweep voltammograms LSV of different concentrations of fenitrothion pesticide in the predicted range of (1 μM–15 μM), on (the modified CPE with safranine layer, 12 cycles), in 0.1M PBS at pH.6, sweep rate 100 mV/s, accumulation time equal to 140 s, and potential window (− 0.4: − 1 V) have been recorded as shown in Fig. 12A.

The calibration curve was constructed and a well fitted linear relationship of the peak current i_p_/μA corresponding to the concentrations of fenitrothion pesticide C/μM was observed with R^2^ parameter equal to 0.99906 as shown in Fig. 12B.

The corresponding regression equation was:

1 $$ {\text{i}}_{{\text{p}}} \left( {\mu {\text{A}}} \right) = {16}.{\text{25138 C}}\left( {\mu {\text{M}}} \right) + { 239}.{899}25138 $$

According to the predicted linearity range mentioned in this study, the limits of detection LOD, and quantification LOQ were calculated. Wenzl et al.^25^ reported the equations of estimated LOD and LOQ in their residual analysis study of food. Three methods of estimating detection and quantification limits are used in the literature. Namely classified into (signal-to-noise, blank determination, and linear regression method)^26^. In our current study the linear regression method was adopted. Where the previously constructed linear calibration curve was expressed with the previously mentioned regression Eq. (1). This model was used to compute the LOD and LOQ. Where they are expressed as:

$$ {\text{LOD}} = {3} \times {\text{S}}.{\text{D}}/{\text{m and LOQ}} = {1}0 \times {\text{S}}.{\text{D}}/{\text{m}} $$

where (S.D) is the standard deviation of the response which is depending up on the standard deviation of y-residuals or the standard deviation of y- intercept, and (m) is the slope^27,28^. Hence, LOD and LOQ were calculated to be 0.10276, and 0.34254 μM, respectively. The parameters of the calibration curve showed good fitting linear relationship with R^2^ equal to 0.99906 as shown in Table 1 which ensures the reliability of the calibration curve.

**Table 1:** Calibration curve parameters of LSV voltammograms for serial concentrations of fenitrothion pesticide in the range of (1–15 μM) using the modified CPE with electropolymerization process in 0.1M PBS at pH.6, sweep rate 100 mV/s, accumulation time equal to 140 s, and potential window (− 0.4: − 1 V).

Calibration curve parameters	Calibration curve values
Linearity range (μM)	1–15
Slope/(µA/µM)	16.25138
Intercept / (µA)	239.899
R-Square (COD)	0.99906
Number of Points (N)	10
Standard error (S.E)	0.17604
Standard deviation (S.D)	0.55668
LOD (µM)	0.10276
LOQ (µM)	0.34254

#### Repeatability and reproducibility measurements (Recovery, Accuracy, Precision _r_, Precision _R,_ calculations)

Repeatability and reproducibility measurements for this promisingly developed method were demonstrated by performing 3 replicate measurements for three different fenitrothion concentrations (4, 6, 8 μM) confined in the previously estimated linearity range. The results showed reliable precision limits in between (0.259: 0.854) through one day for repeatability and precision limits in the range (0.296: 0.890) through 3 days for reproducibility measurements as shown in Tables 2 and 3 respectively. Insignificant differences have been observed between measured and taken concentrations. Narrow tolerance and satisfaction levels have been reached for the calculated recovery, accuracy, and precision for the developed LSV method ensuring the reliability and validity of the suggested method.

**Table 2:** Repeatability validation characteristics of different concentrations of fenitrothion pesticide within the linearity range using LS voltammetric developed method.

Repeatability (interday measurements)								
Day	[C, Taken] μM	n	i_p_/μA	[C, Measured] μM	[C, Measured] μM—Mean	Recovery, %R	Accuracy, %RE	Precision _r_, %RSD
1st	4	1	304.9	4.005	3.968	99.2	0.8	0.854
		2	303.9	3.938				
		3	304.3	3.962				
	6	1	338	6.036	6.021	100.35	0.35	0.259
		2	337.8	6.024				
		3	337.5	6.005				
	8	1	370	8.005	7.982	99.775	0.225	0.695
		2	370	8.005				
		3	368.9	7.937

**Table 3:** Reproducibility validation characteristics for different concentrations of fenitrothion pesticide within the linearity range using the LS voltammetric developed method.

Reproducibility—intraday measurements								
Day	[C, Taken] μM	n	i_p_/μA	[C, Measured] μM	[C, Measured] μM—Mean	Recovery, %R	Accuracy, %RE	Precision _R_, %RSD
1st	4	1	303.7	3.92	3.956	98.9	1.1	0.89
		2	304.2	3.96				
		3	304.8	3.99				
	6	1	337.9	6.03	6.053	100.88	0.88	0.34
		2	338.4	6.06				
		3	338.5	6.07				
	8	1	369.2	7.96	7.978	99.73	0.275	0.296
		2	370	8.005				
		3	369.4	7.97				
2nd	4	1	304.1	3.95	3.97	99.25	0.75	0.534
		2	304.6	3.98				
		3	304.8	3.99				
	6	1	338.09	6.04	6.066	101.1	1.1	0.465
		2	338.8	6.09				
		3	338.6	6.07				
	8	1	369.5	7.97	7.993	99.91	0.7	0.314
		2	370.2	8.02				
		3	369.9	7.99				
3rd	4	1	303.98	3.94	3.96	99	1	0.50
		2	304.2	3.96				
		3	304.5	3.98				
	6	1	339	6.09	6.07	101.16	1.16	0.435
		2	338.7	6.08				
		3	338.1	6.04				
	8	1	369.1	7.95	7.966	99.58	0.425	0.463
		2	369.8	7.99				
		3	369.3	7.96				

#### Ruggedness test

The flexibility and reliability of the suggested LSV method toward deliberate variations were investigated. The concentration of safranine aromatic monomer solution was varied between (0.01 mM–0.03 mM) in the construction step of the modified CPE using safranine solution in PB, pH.6, and 12 cycles previously optimized by electropolymerization. Then the determining step was performed by LSV based on three replicate measurements of 4μM fenitrothion solution, a sweep rate 100mV/s, BPS pH.6, a potential window (− 0.4: − 1 V), and an optimized accumulation time 140s. Insignificant differences between the taken and measured concentration values were observed. Furthermore, the mean percentage recovery and precision obtained within the studied variables were insignificantly varied at (98.33–98.93%) and (1.39–2.6%) respectively as shown in Table 4. Therefore, the suggested developed method is valid and reliable for the assay of fenitrothion pesticide in quality control samples and it could be considered as a rugged method.

**Table 4:** Ruggedness measurements for LSV based on three replicate measurements of 4μM fenitrothion solution, sweep rate 100 mV/s, BPS pH.6, potential window (− 0.4: − 1 V), optimized accumulation time 140 s, and the variable was the concentration of safranine aromatic monomer solution which was varied in between (0.01–0.03 mM) in the construction step of the modified CPE.

Variables	[C, Taken] μM	n	i_p_/μA	[C, Measured] μM	[C, Measured] μM–Mean	Recovery, %R	Precision _r_, %RSD
Concentration of safranine solution							
0.01 mM	4 μM	1	303.7	3.93	3.94	98.5	2.6
		2	303.8	3.94			
		3	304.1	3.95			
0.02 mM	4 μM	1	304	3.94	3.933	98.325	2.08
		2	303.9	3.93			
		3	303.9	3.93			
0.03 mM	4 μM	1	304.2	3.96	3.957	98.93	1.39
		2	304	3.94			
		3	304.4	3.97			

#### Determining F-value and t-value using the HPLC standard method vs. LSV developed method.

The validity of the suggested developed LSV method was demonstrated by measuring the variance between two sets of measurements for specific concentrations confined in the previously optimized linearity range using the HPLC standard method compared to the LSV proposed method. The standard HPLC method was stated in the CIPAC handbook, volume N, 35/TC/M3, P.46^29^. The fenitrothion pesticide was determined with normal phase chromatography, UV detection at 268nm, and a stainless-steel column packed with polar stationary phase Zorbax CN, with a porosity 5 μm. The fenitrothion concentration of 8 μM was prepared and determined using both methods. Each measurement was replicated 5 times where n = 5 and the degree of freedom was equal to 4. The means of the found concentration values for LSV and HPLC in both methods were found to be 7.986 μM and 7.988 μM respectively with insignificant differences. The F-value and t-value were calculated at a 95% level of confidence to be 1.482 and 0.123 respectively as shown in table.5. These values don’t exceed the tabulated F-value and t-value of 6.388 and 2.776 ensuring the similarity between the results of the two analytical methods hence the reliability and validity of the suggested LSV method.

**Table 5:** Variance, F-value, and t-value for two sets of measurements of 8μM fenitrothion pesticide using the suggested LSV method and the standard HPLC method.

Method	[C, taken] μM	Mean [C, found] μM		ST. DV (S)	Variance (S)^2^	F-value	*t-value*
LSV Voltammetric method	8	7.96	7.986	0.023	0.000529	1.482	0.123
		7.99					
		7.97					
		8.02					
		7.99					
HPLC Chromatographic method	8	7.98	7.988	0.028	0.000784		
		7.98					
		8.01					
		7.95					
		8.02					

#### Assay of fenitrothion in the commercial formulation Sumithion™ at 50% EC. (Selectivity measurements)

**Figure 13:**
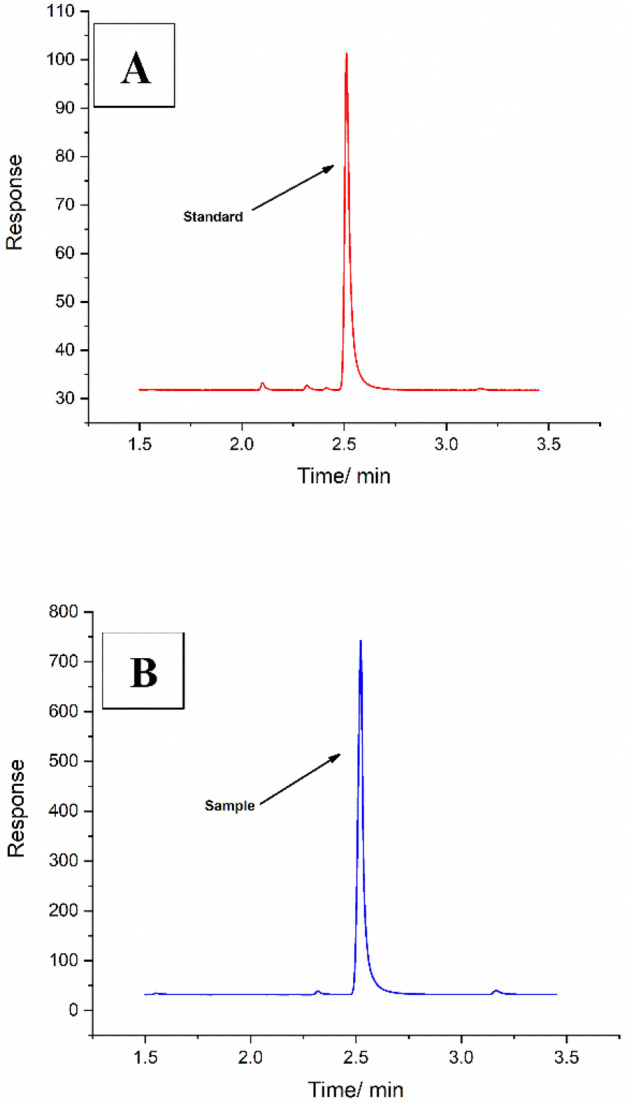
(**A**) Chromatogram of 0.5 mg/L standard fenitrothion, normal phase chromatography, UV detection at 268 nm, oven temperature 40 °C, flow rate 1 ml/min, and stainless-steel column packed with Zorbax-CN (5 μm), (**B**) Chromatogram of 3 mg/L fenitrothion (sumithion50%EC-commercial formulation), normal phase chromatography, UV detection at 268 nm, oven temperature 40 °C, flow rate 1 ml/min, and stainless-steel column packed with Zorbax-CN (5 μm).

The previously suggested LSV method was used to quantify the active ingredient 3content of sumithion™ 50% EC which was collected from the Egyptian market and manufactured by KZ for a pesticide and chemicals company. This recipe includes the fenitrothion active ingredient from Sumitomo chemical Ltd—Japan, emulsifiers which act as predicted interferents. These chemical interferents consists of nonionic polyoxyethylene sorbitol hexaoleate, anionic polyoxyethelene tridecyl diphosphate, and organic solvent. These emulsifiers are blended by 8–10% of the sumithion™ recipe to make the fenitrothion pesticide in the emulsion form when it mixes with water. The previously mentioned standard HPLC method was used to quantify the same sample as shown in Fig. 13A,B. A known concentration of 3 mg fenitrothion active ingredient from Sumithion 50% EC commercial formulation was prepared to be confined in the linearity range. Known concentrations were prepared using fenitrothion certified reference material (CRM) to construct the calibration curves for both methods. The measurements for both the suggested LSV method and the standard HPLC method were replicated 3 times. The mean found concentrations for the desired Sumithion 50% EC sample were found to be 3.018 mg, and 3.043 mg, respectively with satisfaction levels of recovery, and precision.

These obtained results ensured the validity and rigidity of the developed method towards interferents which may be present in the EC commercial formulations of fenitrothion pesticide.

## Conclusion

This work introduced a promising method for the quantification of fenitrothion pesticides based on electrochemically modified CPE with safranine aromatic monomer via an electropolymerization process. The morphology of the surface was investigated via SEM images. The conducting polymer layer on CPE obviously enhanced the reduction peak of the nitro group of fenitrothion pesticide. This was the paradigm to establish this previous method and validate it. The suggested LSV method showed good linearity in the range 1–15 μM, LOD equal to 0.1 μM, and LOQ equal to 0.34 μM with satisfactory levels of recovery, accuracy, and precision. It showed flexibility towards a deliberate variation of the concentration of safranine solution, so it was considered a rugged method. The modified electrode showed very good reproducibility. Repeatedly LS voltammograms of 5µM fenitrothion solution on the modified CPE and the peak current was unchanged for 10 time before it starts to drop gradually. The electroanalytical LSV method vs. the HPLC standard method to quantify the known concentration of fenitrothion showed similar results for two sets of measurements with an F-value and a t-value that did not exceed their tabulated values. Finally, this promising LSV method was successfully used to quantify the fenitrothion active ingredient content in the commercial formulation Sumithion™ 50% EC from Sumitomo Chemicals-Ltd- Japan.

## Data Availability

All data presented in this study are included in this published article.
